# Pre-Transplant Serum Leptin Levels and Relapse of Acute Myeloid Leukemia after Allogeneic Transplantation

**DOI:** 10.3390/ijms23042337

**Published:** 2022-02-20

**Authors:** Mark-Alexander Schwarzbich, Hao Dai, Lambros Kordelas, Dietrich W. Beelen, Aleksandar Radujkovic, Carsten Müller-Tidow, Peter Dreger, Thomas Luft

**Affiliations:** 1 Department of Internal Medicine V, University of Heidelberg, 69120 Heidelberg, Germany; m.schwarzbich@qmul.ac.uk (M.-A.S.); aleksandar.radujkovic@med.uni-heidelberg.de (A.R.); carsten.mueller-tidow@med.uni-heidelberg.de (C.M.-T.); peter.dreger@med.uni-heidelberg.de (P.D.); 2 Department of Epidemiology, German Cancer Research Centre (DKFZ), 69120 Heidelberg, Germany; h.dai@ki.se; 3 Department of Bone Marrow Transplantation, University Hospital, 45122 Essen, Germany; lambros.kordelas@uk-essen.de (L.K.); dietrich.beelen@uk-essen.de (D.W.B.)

**Keywords:** leptin, Adiponectin, allogeneic stem cell transplantation, acute myeloid leukemia, AML, acute lymphoblastic leukemia, ALL, relapse, survival, body mass index

## Abstract

Weight loss and metabolic activity influence outcome after allogeneic stem cell transplantation (alloSCT). This study evaluates pre-conditioning Leptin, a peptide hormone involved in metabolism and immune homeostasis, as a prognostic factor for survival, relapse and non-relapse mortality (NRM) following alloSCT. Leptin serum levels prior to conditioning were determined in a cohort of patients transplanted for various hematologic malignancies (*n* = 524) and correlated retrospectively with clinical outcome. Findings related to patients with acute leukemia (AL) from this sample were validated in an independent cohort. Low pre-conditioning serum Leptin was an independent prognostic marker for increased risk of relapse (but not of NRM and overall mortality) following alloSCT for AL of intermediate and advanced stage (beyond first complete remission). Multivariate analysis revealed a hazard ratio (HR) for relapse of 0.75 per log2 increase (0.59–0.96, *p* = 0.020). This effect was similar in an independent validation cohort. Pre-conditioning serum Leptin was validated as a prognostic marker for early relapse by fitting the multivariate Cox model to the validation data. Pre-conditioning serum Leptin levels may serve as an independent prognostic marker for relapse following alloSCT in intermediate and advanced stage AL patients. Prospective studies are required to prove whether serum Leptin could be used for guiding nutritional intervention in patients with AL undergoing alloSCT.

## 1. Introduction

Allogeneic stem cell transplantation (alloSCT) is an effective treatment for high-risk hematologic malignancies [1,2]. While graft-versus-host disease (GvHD) is known as the main determinant of non-relapse mortality (NRM) after alloSCT [3,4], host factors governing relapse following alloSCT are still poorly understood.

Weight and metabolic status can influence outcome after alloSCT. Numerous studies have suggested that being either overweight or underweight at alloSCT may affect NRM and survival in both adults and children [5,6,7,8,9,10,11,12]. Pre-transplant metabolic status is an important and potentially modifiable predictor for 2-year outcome in those patients [8]. Our group has reported that pre-transplant weight loss and low total serum protein levels independently predicted relapse in patients with acute myeloid leukemia (AML) after alloSCT [13]. Similarly, pre-transplant weight loss was associated with poor disease-free and overall survival in patients allografted for myelodysplastic syndrome (MDS) [14]. 

Leptin is a peptide hormone secreted by adipocytes. Serum levels correlate with total body fat mass [15]. Leptin acts by binding to specific receptors in the hypothalamus and alters expression of several neuropeptides that regulate neuroendocrine function, energy intake and expenditure. Thus, Leptin plays a crucial role in the pathogenesis of obesity and eating disorders and is thought to mediate the neuroendocrine response to food deprivation [16,17,18]. Leptin is also an important regulator of immune homeostasis [19,20].

The known functions of Leptin in metabolism and regulation of immune homeostasis has led us to hypothesize that Leptin levels before alloSCT may be associated with alloSCT outcome.

## 2. Results

### 2.1. Pre-Conditioning Serum Leptin Levels Predict Time to Relapse (TTR) Depending on Disease Entity and Disease Stage

In the discovery cohort, multivariate Cox regression analysis showed a trend towards lower relapse rates with higher serum Leptin levels (HR per log2 increase (95% CI): 0.91 (0.82–1.00); *p* = 0.061; Table S1). Since patients undergoing alloSCT represent a heterogeneous group of disease entities, the interaction factor serum Leptin (pg/mL) * disease entity (AML and ALL) was introduced in the Cox model, indicating a significant interaction of serum Leptin levels and the diagnosis of acute leukaemia (AL) (HR (95% CI): 0.83 (0.71–0.99); *p* = 0.033; Table S2). Accordingly, univariate Cox regression analysis revealed a significant lower risk of relapse with increasing serum Leptin levels in patients with AL, but not in patients with other diagnoses (AL: HR 0.85 (0.72–0.99); *p* = 0.039; other: HR (95% CI): 1.01 (0.89–1.14); *p* = 0.931).

Moreover, focussing our analysis on patients with AL, we identified a significant interaction of serum Leptin levels and disease stage (serum Leptin (pg/mL) × disease stage (intermediate/advance)-HR (95% CI): 0.79 (0.63–0.99), *p* = 0.040) (Table S3). Univariate Cox regression analysis confirmed a reduced risk of relapse with higher Leptin in patients with intermediate (second complete remission, CR2) and advanced stage disease (beyond CR2) only (intermediate/advanced stage: HR (95% CI): 0.81 (0.66–0.99); *p* = 0.048. early stage: HR (95% CI): 0.88 (0.68–1.13); *p* = 0.307). The final training and validation cohorts including acute leukemia > CR1 are represented in Table 1.

### 2.2. Leptin Levels and Outcome in Patients with Intermediate/Advanced Stage AL

For patients with intermediate/advanced stage AL, higher serum Leptin levels were associated with significantly longer TTR (HR per log2 increase (95% CI): 0.75 (0.59–0.96); *p* = 0.020; Table 2) but also with a significantly higher risk of NRM (HR (95% CI): 1.63 (1.15–2.31); *p* = 0.006). In contrast, no significant effect of serum Leptin levels on OS was observed (Table 2). 

**Figure 1:**
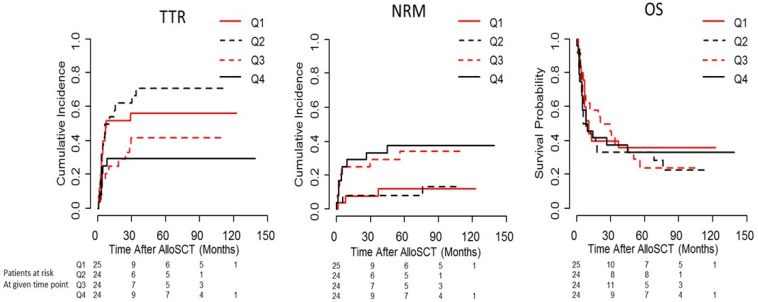
Cumulative incidence function for time to relapse (TTR) and non-relapse mortality (NRM), and Kaplan-Meier curves for overall survival (OS) in quartiles of serum Leptin in intermediate/advanced acute leukaemia of the training cohort (Heidelberg patients). Q1 = 1st quartile of pre-conditioning serum Leptin; Q2 = 2nd quartile of pre-conditioning serum Leptin; Q3 = 3rd quartile of pre-conditioning serum Leptin; Q4 = 4th quartile of pre-conditioning serum Leptin.

To visualize the effect of the continuous variable on the distribution of survival times, the quartiles of serum Leptin levels with regards to TTR, NRM and OS are given in Figure 1. 

**Figure 2:**
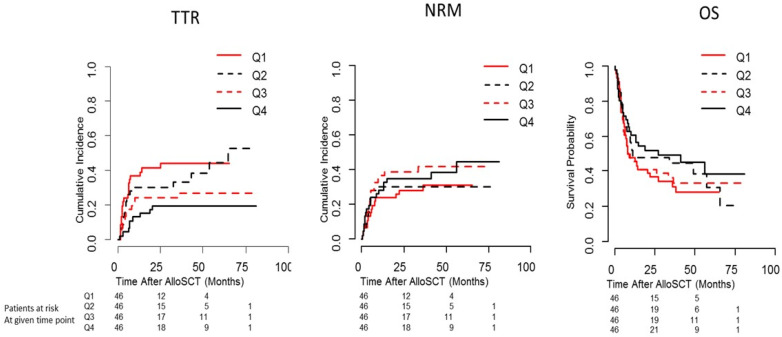
Cumulative incidence function for time to relapse (TTR) and non-relapse mortality (NRM), and Kaplan-Meier curves for overall survival (OS) in quartiles of serum Leptin in intermediate/advanced acute leukaemia of the validation cohort (Essen patients). Q1 = 1st quartile of pre-conditioning serum Leptin; Q2 = 2nd quartile of pre-conditioning serum Leptin; Q3 = 3rd quartile of pre-conditioning serum Leptin; Q4 = 4th quartile of pre-conditioning serum Leptin.

In the independent validation cohort (consisting of patients with AML only), multivariate Cox regression analysis revealed a significant increase of relapse risk with lower serum Leptin levels in patients of intermediate and advanced stage only (serum Leptin (pg/mL) × disease stage (intermediate/advanced)—HR (95% CI): 0.83 (0.72–0.96); *p* = 0.013; Table S4). This was confirmed by applying univariate Cox regression analysis separately on early stage vs. intermediate or advanced stage patients. A reduced risk of relapse with higher serum Leptin was identified among intermediate and advanced stage patients (AML disease stage intermediate/advanced: HR (95% CI): 0.85 (0.74–0.98); *p* = 0.020. AML disease stage early: HR (95% CI): 1.027 (0.86–1.21); *p* = 0.307), results visualized in Figure 2. 

Multivariate Cox regression analysis, when confined to AML patients of intermediate and advanced stages (validation cohort, see Table 1), revealed a significantly reduced risk of relapse with higher pre-conditioning serum Leptin values (HR (95% CI): 0.84 (0.72–0.97); *p* = 0.020). No significant effects on NRM (HR (95% CI):1.09 (0.91–1.31); *p* = 0.359) or OS (HR (95% CI): 0.936 (0.83–1.06), *p* = 0.292) were observed (Table 3). 

**Figure 3:**
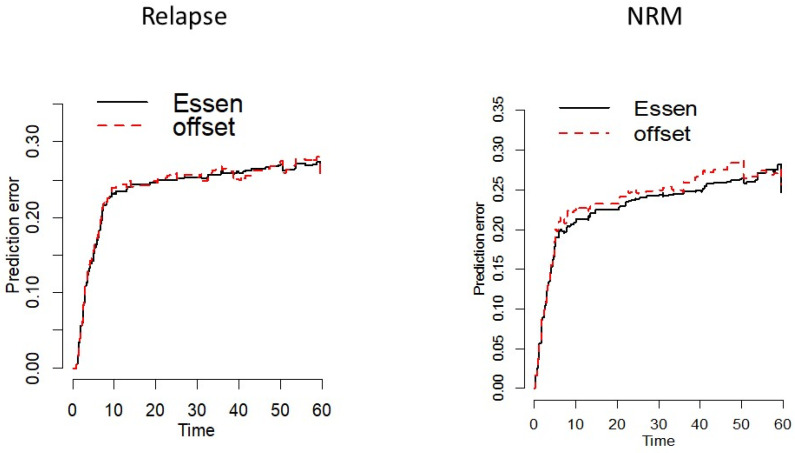
Prediction error analysis of time to relapse (TTR) and non-relapse mortality (NRM) in intermediate/advanced AML of the validation cohort (black line) compared to the training cohort (dashed red line, offset). Comparable prediction errors are shown with the offset and actual Leptin effect from the validation cohort with endpoint TTR, but not with endpoint NRM.

To further validate Leptin as a predictor of relapse in patients with intermediate and advanced stage AML, the multivariate Cox model of the training cohort was fitted to the validation data, including an offset that was equal to the effect of Leptin in the model based on patients with acute leukaemia from the training cohort. The effect of Leptin re-estimated in addition to the offset was not significantly different from 1 (HR (95% CI): 1.12 (0.96–1.30), *p* = 0.151), validating similar effects in both cohorts. Lower prediction errors and higher c-Index supported the predictive value of Leptin in both cohorts (Figure S1). Moreover, we were able to demonstrate comparable prediction errors with the offset and actual Leptin effect from the validation cohort with endpoint TTR but not with endpoint NRM (Figure 3). 

### 2.3. Correlations of Leptin with BMI, Pre-Transplant Weight Change, and Adiponectin 

In the cohort of the Heidelberg AL patients, Leptin serum levels correlated with BMI (r_s_(162) = 0.505, *p* < 0.001) but not with weight change (r_s_(141) = 0.0759, *p* = 0.371) and Adiponectin serum levels (r_s_(167) = 0.0840, *p* = 0.279). Adiponectin showed a negative correlation with BMI (r_s_(88) = −0.220, *p* = 0.014). No association with TTR in the higher risk AL cohort was found for Adiponectin in multivariate Cox analyses including Leptin (Table S5). When BMI is included into the multivariate Cox regression (*n* = 81, cut 22 kg/m^2^ and 30 kg/m^2^), the association of serum Leptin levels with time to relapse also remained significant (Table S6).

In the Essen cohort, only BMI was available. Again, a correlation of serum Leptin levels with BMI could be observed (r_s_(338) = 0.5456, *p* < 0.001). 

### 2.4. Relationship of Serum Leptin Levels and BMI with Disease Stage

**Figure 4:**
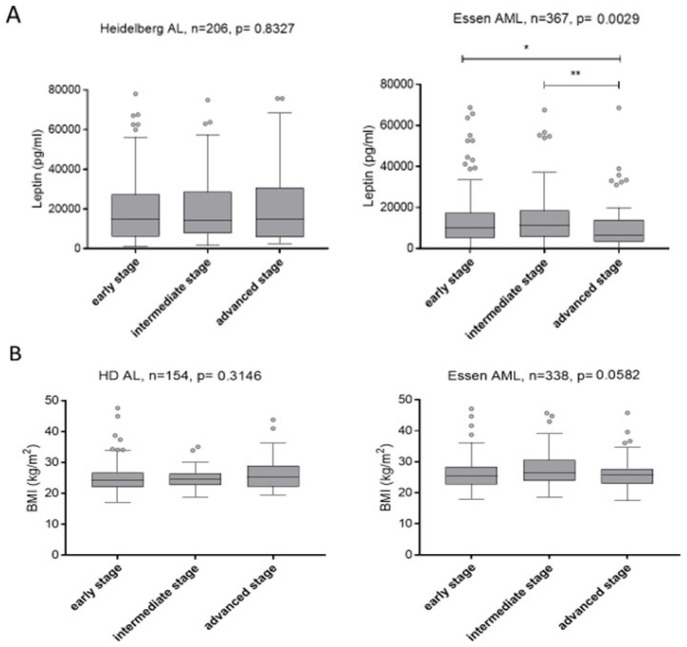
(**A**): Comparison of serum Leptin levels between early, intermediate and advanced stage patients in the Heidelberg acute leukemia (AL) cohort, and the Essen acute myeloid leukemia (AML) cohort (**B**): Comparison of BMI between early, intermediate and advanced stage patients of the Heidelberg acute leukemia (AL) and Essen acute myeloid leukemia (AML) cohorts. Significance code: ‘**’ *p* < 0.01; ‘*’ *p* < 0.05.

In the Heidelberg AL cohort, serum Leptin levels did not differ in patients of various disease stages (*p* = 0.833), whereas in the Essen cohort, serum Leptin levels were significantly lower in advanced stage patients (median 7209 pg/mL, range 369–92,219 pg/mL) compared to early and intermediate stage patients (median 10,790 pg/mL, range 92–68747 pg/mL) (*p* = 0.003) (Figure 4). BMI was not significantly associated with disease stage in both cohorts, Heidelberg (*p* = 0.315) and Essen (*p* = 0.058) (Figure 4). Patients in both cohorts and of any disease stage were within or above the normal range of BMI with a median of 24.2 kg/m^2^, 24.5 kg/m^2^, 25.3 kg/m^2^ for early, intermediate and advanced stage patients of the Heidelberg cohort and 25.5 kg/m^2^, 26.4 kg/m^2^ and 25.7 kg/m^2^ for early, intermediate and advanced stage patients in the Essen cohort respectively. 

### 2.5. Influence of Serum Leptin Levels on Incidence and Severity of Acute and Chronic GvHD

Multivariate Cox-regression analysis of Leptin and known covariables neither showed any significant influence on risk of overall acute GvHD, nor on risk of acute GvHD grades 3 and 4 among patients in the training cohort (Table S7) or in the validation cohort (Table S8). 

Data on chronic GvHD were available for training cohort patients only. Multivariate Cox-regression analysis of pre-conditioning serum Leptin levels and known covariable revealed no significant influence on risk of mild (*p* = 0.373) or severe chronic GvHD (*p* = 0.721) (Table S9).

## 3. Discussion

Relapse remains the most common cause of adverse outcome after alloSCT for AML [22]. A known risk factors for AML relapse include percentage of blasts in the bone marrow and disease status prior to alloSCT, conditioning intensity as well as cytogenetics [23,24,25]. Here, low pre-conditioning serum Leptin levels are identified to be a prognostic marker of early relapse in Acute Leukemia patients of intermediate and advanced stage disease following alloSCT. Moreover, this effect has been validated in an independent cohort of AML patients transplanted at a different centre. 

Our finding is in line with our previous reports showing that the pre-conditioning metabolic status is a risk factor for early relapse following alloSCT for myeloid hematologic malignancies [13]. Since serum Leptin levels correlate with total body fat mass [15], this may point to an overall metabolic influence on the risk of relapse. The impact of pre-transplantation nutritional status on outcome after alloSCT has been well documented, and both positive and negative effects of higher weight prior to transplantation have been noted [5,6]. It has been suggested that reduced OS following alloSCT in overweight children relates to increased rates of treatment-related toxicity [9]. Higher mass of adipose tissue may result in increased volumes of distribution and reduced clearance for lipophilic drugs such as busulfan and cyclophosphamide, which are commonly used in pre-transplant conditioning for allSCT, and thus may possibly result in higher cumulative drug doses and longer drug exposure times [26]. Similarly, we found a significant increase in NRM with higher pre-conditioning Leptin serum levels in the training cohort. However, this effect could not be validated in the independent validation cohort, despite a close correlation between Leptin levels and BMI. 

On the other hand, Medeiros et al. have reported higher rates of complete remission and lower rates of treatment resistance following chemotherapy in obese adult AML patients [10]. Moreover, Yang et al. have reported an increase in OS in adult patients with a BMI of ≥23 kg/m^2^ compared to those with lower BMI following alloSCT [11]. It should be noted that low BMI and malnutrition are well established risk factors for poor prognosis in cancer patients, including those with hematologic malignancies: malnutrition has been linked to higher rates of adverse events following the application of cytostatic drugs, higher rates of infection, delayed wound healing and poor treatment compliance in cancer patients [27]. 

One may assume that higher risk AML patients would be more prone to pre-conditioning weight loss and thus have lower serum Leptin levels because they would require more chemotherapy lines to achieve remission. However, we have previously reported that neither cytogenetics, nor duration from initial diagnosis to transplantation, number of prior chemotherapy cycles or quality of blast response was correlated with weight loss [13]. Moreover, patients displaying a pronounced weight loss of >2% had a median BMI of 25 kg/m^2^ [13]. In the current study, patients in both the training and validation cohort of any disease stage were well within or even above the normal BMI range on average, with similar average BMI in patients of various disease stages. Importantly, serum Leptin levels did not correlate with metabolic markers other than BMI. This indicates that even mild systemic energy deprivation reflected by decreased Leptin serum levels (as opposed to outright malnutrion) may lead to an increased risk of early relapse. 

It is currently unclear why such a state of energy deprivation may contribute to relapse. Nutritional status can affect immune responses and survival in non-hematologic diseases such as HIV [28]. In terms of both structure and function, Leptin resembles IL-6 and is a member of the cytokine superfamily. It stimulates production of pro-inflammatory mediators like IL-1, IL-6, IL-12 and TNF and promotes production of reactive oxygen species, phagocytosis and secretion of prostaglandins and nitric oxide [19]. Activating effects on both innate and adaptive immunity have been described [19,20,29,30]. Leptin has been implicated in promotion of auto-aggressive processes and autoimmune disease [31] ranging from thyroid dysfunction [32], rheumatoid arthritis [33,34,35], Lupus erythematodes [36,37], autoimmune encephalitis [38] and inflammatory bowel disease [39]. An elevation of Leptin was also described following alloSCT, particularly in those patients suffering from chronic graft versus host disease (cGvHD) [40,41]. Starvation-induced Leptin deficiency markedly suppresses cell mediated immune responses [42]. Thus, low serum Leptin levels may negatively affect graft-versus-leukemia (GVL) effects resulting in increased rates of relapse following alloSCT. 

GvL responses often coincide with GvHD, with strongest statistical associations reported for chronic GvHD [43,44]. Interestingly, the current study did not demonstrate any effect of pre-conditioning serum Leptin on risk of either acute or chronic GvHD. It may thus be questioned whether the increased risk of relapse with lower pre-conditioning serum Leptin levels is due to an influence on GvL. However, GvHD-independent GvL effects have been described [45,46], although the biological mechanisms separating GvHD from GvL are still poorly understood. 

Leptin receptor expression itself has been demonstrated on the surface of both ALL [47] and AML [48] cells. Another possible explanation for higher risk of relapse with low serum Leptin levels is tumour dormancy induced by energy deficiency. Energy deprivation resulted in loss of cycling (“hibernation”) in a small number of stem cells in a non-hematopoietic stem cell model [49]. Similarly, hematopoietic stem cells have been shown to react to energy deficiency by activation of liver kinase B1, which in turn induces stem cell quiescence and exit from cell cycle [50,51]. One could thus speculate that energy deprivation results in quiescence of a fraction of leukemia cells that might escape conditioning chemotherapy. Interestingly, we found that low pre-conditioning serum Leptin predicts early relapse in AML patients of intermediate or advanced stages, i.e., in those patients who have relapsed after previous lines of treatment or those who failed to achieve remission before transplantation. In a disease that has demonstrated poor chemosensitivity, even minor systemic energy deprivation may result in additional protection from conditioning treatment of individual leukemic cells located in bone marrow niches, and thus foster subsequent relapse. In chemo sensitive disease, similar levels of energy deficiency may not suffice to protect leukemic cells.

The importance of late immune effects in long-term disease control following alloSCT is evidenced by the association of cGvHD but not aGvHD with reduced risk of relapse [43,45,52,53,54]. As acute leukemias tend to relapse early compared to more chronic hematologic diseases, chemosensitivity and governing metabolic factors may be of greater importance for AML. Moreover, in diseases of lymphatic origin, therapeutic antibodies such as Rituximab, Blinatumomab or Brentuximab are commonly used. Those substances target malignant cells irrespective of cell cycling, which may explain why the observed effect is limited to AML patients. Further experimental evidence is needed to substantiate hypotheses related to either influence on GvL effects or leukemia cell quiescence.

When including BMI or Adiponectin together with Leptin into the Cox regression, an independent, significant, prognostic effect of Leptin in higher risk acute leukemia remained, underlining the novel and is a unique characteristic of this marker.

Potential limitations of this study include its retrospective nature, heterogeneity in patient characteristics, differences in pre-transplant and conditioning treatment as well as sample size. 

## 4. Patients and Methods

### 4.1. Patient Characteristics

Patients allografted for both myeloid and lymphoid hematologic malignancies were eligible for this study if they had undergone alloSCT at the University Hospital Heidelberg between 2002 and 2013 and had serum samples available for measurement of pre-conditioning Leptin (discovery cohort, *n* = 524; Table S10). The observed effects were confirmed in an independent cohort of patients with AML who had been allografted at the Department of Bone Marrow Transplantation of the University Hospital Essen (Germany) between 2009 and 2013 (*n* = 367; Table S11). Written informed consent to sample and data collection according to the Declaration of Helsinki was obtained from all patients, and the local ethics committees of both institutions approved the study (Vote 120/2002, 18 June 2018).

The training cohort comprised patients with acute leukaemia (AML and ALL) of an intermediate or advanced stage (beyond first complete remission, >CR1) from the full Heidelberg cohort (*n* = 97). The validation cohort consisted of patients with AML of intermediate or advanced stage who underwent allografting in Essen (*n* = 184). Patient characteristics of both the training and validation cohort are shown in Table 1. For collection of serum and clinical data, written informed consent according to the Declaration of Helsinki was obtained for all patients, and the local ethics committees approved sample and data collections. Patient data were obtained from medical records and chart review. Disease stage before alloSCT was assessed applying published criteria: for AML and ALL: low = CR1, intermediate = CR2, advanced = all others [52]. Acute and chronic GvHD were diagnosed clinically and histologically and graded based on established standard criteria [55,56]. Data on chronic GvHD were available for patients of the training cohort only.

### 4.2. Determination of Serum Leptin and Adiponectin Levels

Serum samples were collected prospectively at the final pre-transplantation visit 2 to 4 weeks before starting conditioning therapy. Serum levels of Leptin and Adiponectin were quantified retrospectively in eligible patients by ELISA using the human Leptin and the human Adiponectin/Acrp30 DuoSet (both R&D Systems, Minneapolis, MN, USA).

### 4.3. Transplantation Procedure and Supportive Care

AlloSCT was performed according to local standard operating procedures. GVHD prophylaxis and supportive care were performed as previously described [57,58]. All Leptin serum and Adiponectin levels were assessed retrospectively in the same academic laboratory (University Hospital Heidelberg, Heidelberg, Germany). GvHD prophylaxis was performed as previously described [58,59].

### 4.4. Statistical Methods

Categorical data were compared using Fisher’s exact test. Continuous variables were compared applying the Kolmogorow-Smirnow test or Kruskal-Wallis test. The relationship between variables was analysed using Spearman’s rank correlation. Distribution of serum Leptin levels was highly skewed and thus log2-transformation was applied. Hazard ratios (HR) for Leptin as a continuous variable are given per log2 increase. NRM and relapse were considered as competing events. Prognostic impact of pre-conditioning Leptin levels on time to relapse (TTR), NRM and overall survival (OS) was evaluated based on hazard ratios (HRs) with 95% CIs from corresponding (cause-specific) Cox regression models using log2-transformed Leptin serum levels as a continuous variable. Multivariable (cause-specific) Cox regression models were used to adjust the effects for additional covariables: Patient age at transplantation, HLA match, conditioning intensity, anti-thymocyte globulin (ATG) GvHD prophylaxis, recipient sex and donor sex. Cumulative incidence of relapse and NRM were analysed by using cumulative incidence curves to account for competing risks and the Kaplan–Meier method was used to estimate OS. To confirm and validate low pre-conditioning Leptin serum levels as a risk factor for relapse, the Cox model was fitted to the validation data, including an offset that was equal to the effect of Leptin deficiency in the model with the training cohort. Calculations were performed using the statistical software environment R (version 3.4.3). All statistical tests were two-sided. HRs were estimated with 95% CIs. Values of *p* < 0.05 were considered statistically significant.

## 5. Conclusions

In conclusion, our results provide additional evidence for an interaction of nutritional homeostasis and control of myeloid malignancies after alloSCT and suggest a potential role of Leptin in this process. It remains to be shown by prospective studies if serum Leptin and/or other metabolic biomarkers could be used for guiding nutritional intervention in patients with AML undergoing alloSCT with the aim of improving outcome.

## Figures and Tables

**Table 1:** Patient characteristics of training cohort (Heidelberg, AL only, intermediate and advanced stage (>CR1) *) and validation cohort (Essen, AML only, intermediated and advanced stage (>CR1) *).

	Heidelberg (Training Cohort)	Essen (Validation Cohort)	*p*
*n*	97	184	NA
Year alloSCT performed	2002–2013	2009–2013	NA
Median Age at alloSCT (range)	56 (19–70)	58 (17–73)	0.149
Recipient Sex Female Male	38 (39%) 59 (61%)	90 (49%) 94 (51%)	0.132
Donor Sex Female Male	22 (23%) 75 (77%)	69 (38%) 115 (62%)	0.016
Disease entity AML ALL	86 (89%) 11 (11%)	184 (100%)	NA
HLA match Match (10/10) Mismatch	66 (68%) 31 (32%)	124 (67%) 60 (33%)	>0.999
Conditioning MAC RIC	16 (16%) 81 (84%)	25 (14%) 159 (86%)	0.594
Pre-transplant ATG Given Not given	75 (77%) 22 (23%)	92 (50%) 92 (50%)	<0.001
Disease score before alloSCT * 1 2	31 (32%) 66 (68%)	97 (53%) 87 (47%)	0.005
Serum Leptin Median in pg/mL (range)	14,924 (1518–90,212)	8581 (91.62–92,219)	0.001
Serum Adiponectin Median in ng/mL (range)	4971.2 (1080–20,000)	NA	

* According to [21]. Abbreviations: ALL, acute lymphocytic leukemia; AML, acute myeloid leukemia; ATG, anti-thymocyte globulin; HLA, human leukocyte antigen; MAC, Myeloablative conditioning; RIC, reduced intensity conditioning.

**Table 2:** Multivariate Cox regression analysis of pre-conditioning serum Leptin levels (pg/mL) and established prognostic covariates with endpoints time to relapse (TTR), non-relapse mortality (NRM) and overall survival (OS) in intermediate/advanced Acute leukaemia (>CR1) *, training cohort.

	TTR		NRM		OS	
	HR (95% CI)	*p*	HR (95% CI)	*p*	HR (95% CI)	*p*
Pre-conditioning Leptin (per log2 increase)	0.75 (0.59–0.96)	0.020	1.63 (1.15–2.31)	0.006	1.05 (0.87–1.28)	0.589
Age at transplant (per 10 years)	1.11 (0.87–1.40)	0.411	1.20 (0.83–1.74)	0.329	1.14 (0.93–1.38)	0.201
HLA match match (10/10) mismatch	Ref 1.39 (0.79–2.63)	Ref 0.187	Ref 1.27 (0.48–3.23)	Ref 0.635	Ref 1.37 (0.79–2.44)	Ref 0.264
Conditioning MAC RIC	Ref 0.72 (0.38–1.26)	Ref 0.310	Ref 1.04 (0.43–2.51)	Ref 0.936	Ref 0.93 (0.56–1.55)	Ref 0.774
ATG Not given Given	Ref 0.46 (0.22–1.04)	Ref 0.060	Ref 0.55(0.17–1.78)	Ref 0.320	Ref 0.48 (0.25–0.94)	Ref 0.034
Recipient sex Female Male	Ref 0.66 (0.34–1.29)	Ref 0.225	Ref 1.88 (0.73–4.84)	Ref 0.188	Ref 1.18 (0.69–2.01)	Ref 0.555
Donor sex Female Male	Ref 0.38 (0.19–0.77)	Ref 0.007	Ref 0.52 (0.17–1.57)	Ref 0.248	Ref 0.42 (0.23–0.77)	Ref 0.005

* According to [21]. Abbreviations: ATG, anti-thymocyte globulin; HLA, human leukocyte antigen; MAC, Myeloablative conditioning; RIC, reduced intensity conditioning, Ref, reference value (=1).

**Table 3:** Multivariate Cox regression analysis of pre-conditioning serum Leptin levels (pg/mL) and established prognostic covariates with endpoints time to relapse (TTR), non-relapse mortality (NRM) and overall survival (OS)—intermediate/advanced stage AML (>CR1) *, validation cohort.

	TTR		NRM		OS	
	HR (95% CI)	*p*	HR (95% CI)	*p*	HR (95% CI)	*p*
Pre-conditioning Leptin (per log2 increase)	0.84 (0.72–0.97)	0.020	1.09 (0.91–1.31)	0.359	0.94 (0.83–1.06)	0.292
Age at transplant (per 10 years)	0.95 (0.76–1.17)	0.607	1.21 (0.96–1.51)	0.100	1.08 (0.93–1.27)	0.315
HLA match match (10/10) mismatch	Ref 1.69 (0.95–3.03)	Ref 0.075	Ref 2.13 (1.26–3.62)	Ref 0.005	Ref 2.14 (1.43–3.21)	Ref <0.001
Conditioning MAC RIC	Ref 1.48 (0.64–3.43)	Ref 0.361	Ref 1.84 (0.66–5.15)	Ref 0.243	Ref 1.77 (0.89–3.55)	Ref 0.106
ATG Not given given	Ref 0.98 (0.56–1.71)	Ref 0.946	Ref 0.57 (0.34–0.96)	Ref 0.033	Ref 0.69 (0.47–1.02)	Ref 0.061
Recipient sex Female Male	Ref 0.71 (0.41–1.23)	Ref 0.218	Ref 0.92 (0.54–1.57)	Ref 0.771	Ref 0.70 (0.47–1.05)	Ref 0.086
Donor sex Female Male	Ref 1.12 (0.62–2.01)	Ref 0.711	Ref 0.52 (0.17–1.57)	Ref 0.248	Ref 0.42 (0.23–0.77)	Ref 0.005

* According to Gratwohl et al., 2009 [21]. Abbreviations: ALL, acute lymphocytic leukemia; AML, acute myeloid leukemia; ATG, anti-thymocyte globulin; HLA, human leukocyte antigen; MAC, Myeloablative conditioning; RIC, reduced intensity conditioning. Ref, reference value (=1).

## Supplementary Materials

The following supporting information can be downloaded at: https://www.mdpi.com/article/10.3390/ijms23042337/s1.
