# Supplementary material for: Pre-Transplant Serum Leptin Levels and Relapse of Acute Myeloid Leukemia after Allogeneic Transplantation
Source: Int J Mol Sci. 2022 Feb 20;23(4):2337. doi: 10.3390/ijms23042337 (PMC8879842; doi:10.3390/ijms23042337)
Supplement: Supplementary file 1 [file ijms-23-02337-s001.zip › ijms-1587461-supplementary.pdf]

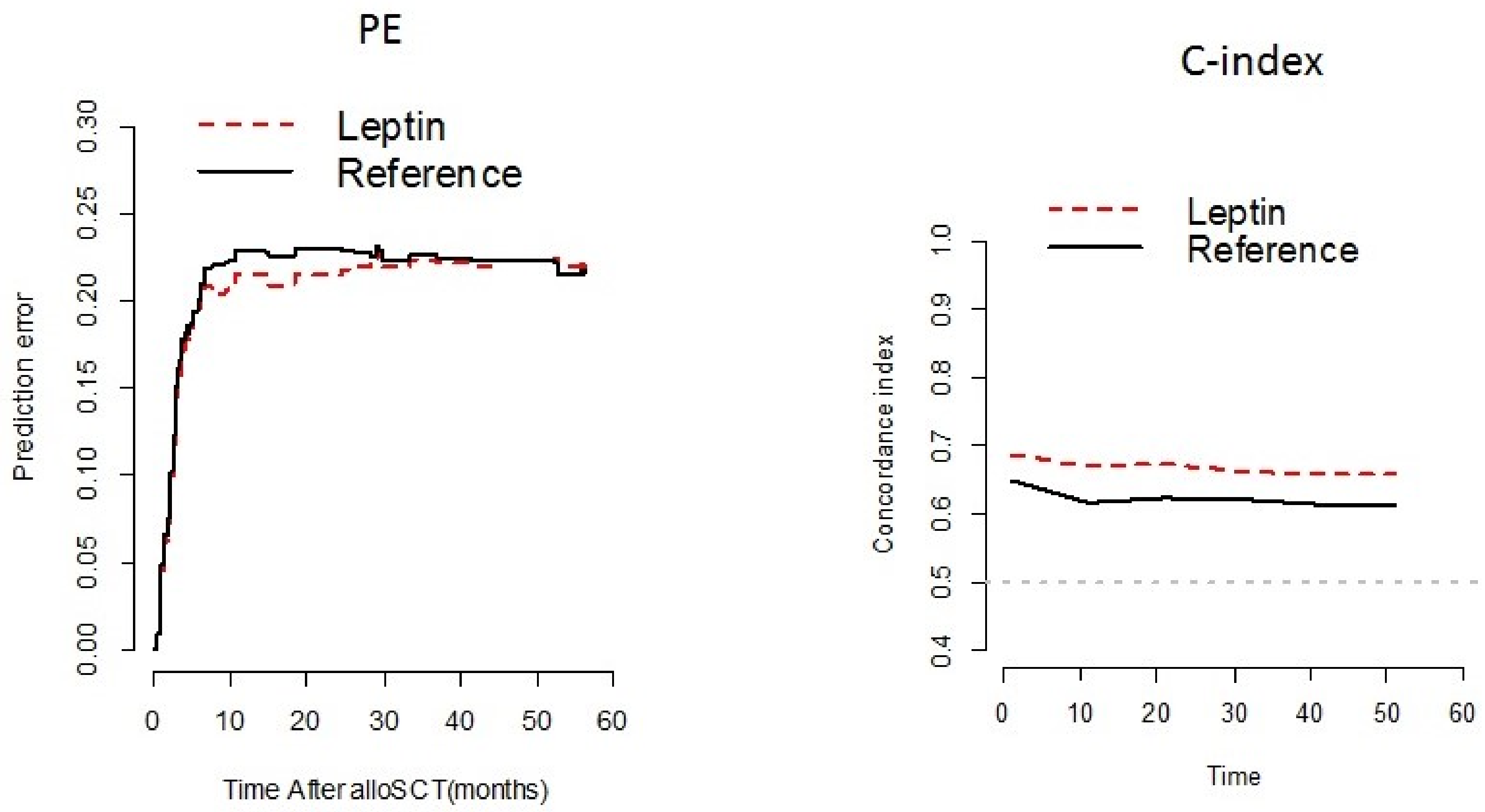

Supplementary Figure S1. *Prediction error analysis and C-index of time to relapse (TTR) prediction in the multivariate model of Heidelberg intermediate/advanced stage Acute Leukaemia patients with and without (reference) consideration of Leptin*

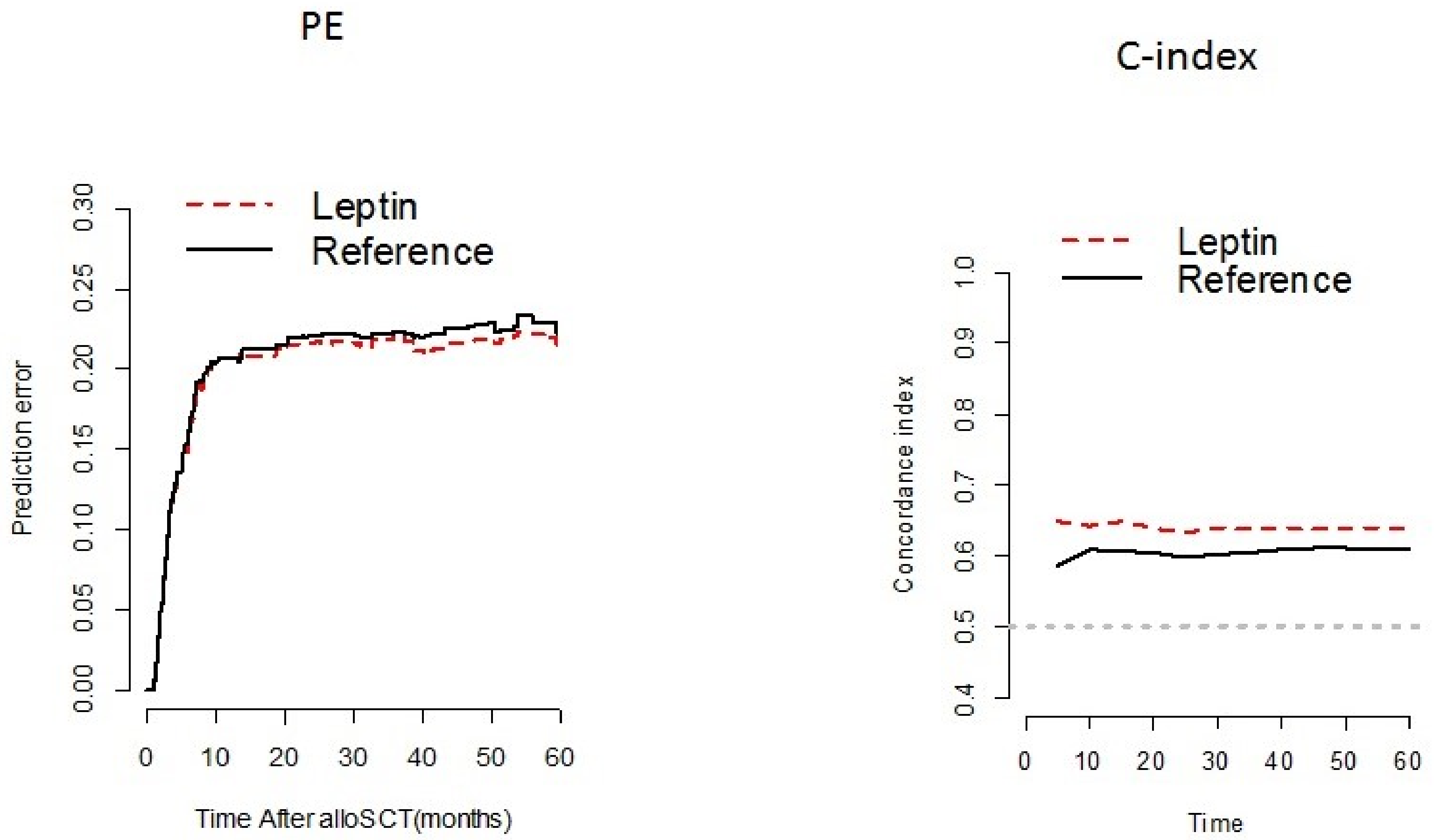

Supplementary Figure S2. *Prediction error analysis and C-index of time to relapse (TTR) prediction in multivariate model of Essen intermediate/advanced stage AML patients with and without (reference) consideration of Leptin.*

Supplementary Table S1. Multivariate Cox regression analysis with endpoint time to relapse (TTR) in the full Heidelberg cohort.

	HR (95% CI)	P	SIG
**Pre-conditioning Leptin (per log2)**	0.91 (0.82 -1.00)	0.061	.
**Age at transplant (per 10 years)**	0.94 (0.83 -1.06)	0.326	
**Disease stage**[+]			
CR1	Ref	Ref	***
>CR1	3.09 (2.11- 4.53)	<0.001	
**Disease entity**			
AML/ALL	Ref	Ref	***
Other	0.57 (0.41 -0.79)	<0.001	
**HLA match**			
match (10/10)	Ref	Ref	
mismatch	0.76 (0.51- 1.12)	0.161	
**Conditioning**			
MAC	Ref	Ref	
RIC	1.22 (0.79- 1.98)	0.367	
**ATG**			
Not given	Ref	Ref	
given	0.92 (0.66 – 1.27)	0.608	
**Recipient sex**			
Female	Ref	Ref	
Male	0.88 (0.64- 1.20)	0.416	
**Donor sex**			
Female	Ref	Ref	
Male	0.90 (0.65- 1.24)	0.512	

*Significance code: '***' p< 0.001; '**' p<0.01; '*' p<0.05; '.' p<0.1; [+] According to Gratwohl et al., 2009 [51]. Abbreviations: SIG-significance; HR-hazard ratio; HLA-human leukocyte antigen; RIC-reduced intensity conditioning; MAC-Myeloablative conditioning; ATG-anti-thymocyte globulin; CR – complete remission before alloSCT.*

Supplementary Table S2. Multivariate Cox regression analysis with endpoint to time to relapse (TTR) in the full Heidelberg cohort including interaction term of disease entity and Leptin serum level.

	HR (95% CI)	P	SIG
**Age at transplant (per 10 years)**	0.94 (0.83 -1.07)	0.344	
**Disease stage**$^{+}$			
CR1	Ref	Ref	***
>CR1	2.99 (2.04- 4.37)	<0.001	
**HLA match**			
match (10/10)	Ref	Ref	
mismatch	0.78 (0.53- 1.15)	0.212	
**Conditioning**			
MAC	Ref	Ref	
RIC	1.20 (0.78- 1.87)	0.406	
**ATG**			
Not given	Ref	Ref	
given	0.92 (0.66 – 1.27)	0.519	
**Recipient sex**			
Female	Ref	Ref	
Male	0.87 (0.64 – 1.19)	0.416	
**Donor sex**			
Female	Ref	Ref	
Male	0.90 (0.65- 1.24)	0.500	
**Leptin (log2) * disease entity (AML and ALL)**	0.83 (0.71 -0.99)	0.033	*
**Leptin (log2) * disease entity (other)**	0.95 (0.84 – 1.09)	0.480	

*Significance code: '***' p< 0.001; '**' p<0.01; '*' p<0.05; '.' p<0.1; * According to Gratwohl et al., 2009 (21). Abbreviations: SIG-significance; HR-hazard ratio; HLA-human leukocyte antigen; RIC-reduced intensity conditioning; MAC-Myeloablative conditioning; ATG-anti-thymocyte globulin. CR – complete remission before alloSCT, AML, acute myeloid leukemia, ALL, acute lymphoid leukemia.*

Supplementary Table S3. Multivariate Cox regression analysis with endpoint time to relapse (TTR) in Acute Leukemia patients, Heidelberg cohort.

	**HR (95% CI)**	**P**	**SIG**
**Age at transplant (per 10 years)**	1.02 (0.85 -1.11)	0.825	
**HLA match**			
match (10/10)	Ref	Ref	
mismatch	0.70 (0.39- 1.28)	0.251	
**Conditioning**			
MAC	Ref	Ref	
RIC	0.85 (0.50- 1.43)	0.530	
**ATG**			
Not given	Ref	Ref	
given	1.28 (0.74 – 2.21)	0.378	
**Recipient sex**			
Female	Ref	Ref	
Male	0.91 (0.55 – 1.52)	0.726	
**Donor sex**			
Female	Ref	Ref	*
Male	0.55 (0.32- 0.93)	0.027	
**Leptin (log2) x disease stage[+] (early)**	0.85 (0.65 -1.12)	0.256	
**Leptin (log2) x disease stage[+] (intermediated/advanced)**	0.79 (0.63 – 0.99)	0.040	*

*Significance code: '***' p< 0.001; '**' p<0.01; '*' p<0.05; '.' p<0.1; [+] According to Gratwohl et al., 2009 (21). Abbreviations: SIG-significance; HR-hazard ratio; HLA-human leukocyte antigen; RIC-reduced intensity conditioning; MAC-Myeloablative conditioning; ATG-anti-thymocyte globulin.*

Supplementary Table S4. Multivariate Cox regression analysis with endpoint time to relapse (TTR) in Acute Leukemia patients, Essen cohort, considering interaction of disease stage and Leptin serum level.

	HR (95% CI)	*p*	*SIG*
**Age at transplant (per 10 years)**	1.00 (0.85 -1.18)	0.995	
**HLA match**			
match (10/10)	Ref	Ref	
mismatch	1.34 (0.73-2.63)	0.163	
**Conditioning**			
MAC	Ref	Ref	
RIC	1.38 (0.73- 2.63)	0.320	
**ATG**			
Not given	Ref	Ref	
given	0.85 (0.57 – 1.28)	0.441	
**Donor sex**			
Female	Ref	Ref	
Male	0.79 (0.53- 1.18)	0.252	
**Recipient sex**			
Female	Ref		
Male	1.01 (0.67-1.52)	0.973	
**Leptin (log2) x disease stage⁺ (early)**	1.02 (0.86 -1.21)	0.813	
**Leptin (log2) x disease stage⁺ (intermediate/advanced)**	0.83 (0.72 – 0.96)	0.013	*

*Significance code: '***' p< 0.001; '**' p<0.01; '*' p<0.05; '.' p<0.1; ⁺ According to Gratwohl et al., 2009 (34). Abbreviations: SIG-significance; HR-hazard ratio; HLA-human leukocyte antigen; RIC-reduced intensity conditioning; MAC-Myeloablative conditioning; ATG-anti-thymocyte globulin.*

Supplementary Table S5. Multivariate Cox regression analysis **including Adiponectin** (n=85), with endpoint time to relapse (TTR) in intermediate/advanced Acute leukaemia (>CR1)#, training cohort

	**HR (95% CI)**	**P**	**SIG**
Pre-conditioning Adiponectin (per log2)	0.84 (0.60-1.19)	0.331	
Pre-conditioning Leptin (per log2)	0.73 (0.57-0.95)	0.018	*
Age at transplant (per 10 years)	1.02 (0.99-1.06)	0.136	
HLA match			
match (10/10)	Ref		
mismatch	1.05 (0.71-1.57)	0.794	
Conditioning			
MAC	Ref		
RIC	1.33 (0.66-2.69)	0.427	
ATG			
Not given	Ref		
given	0.62(0.27-1.44)	0.269	
Donor sex			
Female	Ref		
Male	0.57 (0.27-1.44)	0.164	
Recipient sex			
Female	Ref		
Male	0.51 (0.25-1.05)	0.069	

*Significance code: '***' p< 0.001; '**' p<0.01; '*' p<0.05; '.' p<0.1, # According to Gratwohl et al., 2009 (34). Abbreviations: SIG-significance; HR-hazard ratio; HLA-human leukocyte antigen; RIC-reduced intensity conditioning; MAC-Myeloablative conditioning; ATG-anti-thymocyte globulin.*

Supplementary Table S6. Multivariate Cox regression analysis **including BMI** (n=81), with endpoint time to relapse (TTR) in intermediate/advanced Acute leukaemia (>CR1)[#], training cohort

	HR (95% CI)	*p*	*SIG*
BMI <22 kg/m^2	Ref		
BMI 22-29.9 kg/m^2	0.62 (0.13-3.22)	0.591	
BMI≥30 kg/m^2	1.48 (0.55-3.97)	0.432	
Pre-conditioning Leptin (per log2)	0.70 (0.51-0.98)	0.038	*
Age at transplant (per 10 years)	1.01 (0.98-1.05)	0.553	
HLA match			
match (10/10)	Ref		
mismatch	1.24 (0.83-1.84)	0.286	
Conditioning			
MAC	Ref		
RIC	1.46 (0.45-4.68)	0.527	
ATG			
Not given	Ref		
given	0.31 (0.12-0.83)	0.019	*
Donor sex			
Female	Ref		
Male	0.30 (0.11-0.80)	0.017	*
Recipient sex			
Female	Ref		
Male	0.58 (0.24-1.42)	0.236	

*Significance code: '***' p< 0.001; '**' p<0.01; '*' p<0.05; '.' p<0.1, [#] According to Gratwohl et al., 2009 (34). Abbreviations: SIG-significance; HR-hazard ratio; HLA-human leukocyte antigen; RIC-reduced intensity conditioning; MAC-Myeloablative conditioning; ATG-anti-thymocyte globulin.*

Supplementary Table S7. *Multivariate Cox regression analysis with endpoints acute GvHD and acute GvHD grade 3+4 in* Acute Leukemia patients *(Heidelberg cohort).*

	**Acute GvHD**		**Acute GvHD grade 3+4**	
	**HR (95% CI)**	**P**	**HR (95% CI)**	**P**
**Pre-conditioning Leptin (per log2)**	0.94 (0.72 -1.22)	0.630	0.88 (0.59 -1.33)	0.544
**Age at transplant (per 10 years)**	0.93 (0.74 -1.19)	0.579	1.22 (0.82 -1.83)	0.326
**HLA match**				
match (10/10)	Ref	Ref	Ref	Ref
mismatch	1.15 (0.55 -2.42)	0.706	2.29 (0.76 -6.89)	0.140
**Conditioning**				
MAC	Ref	Ref	Ref	Ref
RIC	0.35 (0.17 -0.70)	0.003 **	0.26 (0.09 -0.77)	0.015 *
**ATG pre transplant**				
Not given	Ref	Ref	Ref	Ref
Given	0.58 (0.26 -1.28)	0.177	0.43 (0.13 - 1.46)	0.176
**Recipient sex**				
Female	Ref	Ref	Ref	Ref
Male	0.58 (0.27 -1.24)	0.160	0.81 (0.26 - 2.52)	0.717
**Donor sex**				
Female	Ref	Ref	Ref	Ref
Male	1.52 (0.65 -3.52)	0.332	1.03 (0.33 - 3.26)	0.959

*Significance code: '***' p< 0.001; '**' p<0.01; '*' p<0.05; '.' p<0.1;* $^{+}$ *According to Gratwohl et al., 2009 (34). Abbreviations: SIG-significance; HR-hazard ratio; HLA-human leukocyte antigen; RIC-reduced intensity conditioning; MAC-Myeloablative conditioning; ATG-anti-thymocyte globulin.*

Supplementary Table S8. *Multivariate Cox regression analysis with endpoints acute GvHD and acute GvHD grade 3+4 in* Acute Leukemia patients *(Essen cohort).*

	**Acute GvHD**		**Acute GvHD grade 3+4**		
	**HR (95% CI)**	**P**	**HR (95% CI)**	**P**	
**Pre-conditioning Leptin (per log2)**	1.13 (0.99 -1.27)	0.052	1.00 (0.73 -1.37)	0.991	
**Age at transplant (per 10 years)**	1.00 (0.86 -1.16)	0.988	0.97 (0.69 -1.37)	0.881	
**HLA match**					
match (10/10)	Ref	Ref	Ref	Ref	
mismatch	1.14 (0.79 -1.65)	0.486	0.92 (0.36 -2.37)	0.861	
**Conditioning**					
MAC	Ref	Ref	Ref	Ref	
RIC	0.84 (0.47 -1.49)	0.544	4.18 (0.51 - 34.28)	0.183	
**ATG pre transplant**					
Not given	Ref	Ref	Ref	Ref	*
Given	0.91 (0.64 -1.29)	0.588	0.33 (0.13 - 0.97)	0.024	
**Recipient sex**					
Female	Ref	Ref	Ref	Ref	
Male	1.10 (0.74 -1.63)	0.636	1.67 (0.67 - 4.17)	0.275	
**Donor sex**					
Female	Ref	Ref	Ref	Ref	
Male	0.82 (0.58 -1.17)	0.281	0.64 (0.28 - 1.47)	0.290	

*Significance code: '***' p< 0.001; '**' p<0.01; '*' p<0.05; '.' p<0.1;* $^{+}$ *According to Gratwohl et al., 2009 (34). Abbreviations: SIG-significance; HR-hazard ratio; HLA-human leukocyte antigen; RIC-reduced intensity conditioning; MAC-Myeloablative conditioning; ATG-anti-thymocyte globulin.*

Supplementary Table S9. *Multivariate Cox regression analysis with endpoint mild chronic GvHD and severe chronic GvHD in* Acute Leukemia patients *(Heidelberg cohort).*

	**Mild chronic GvHD**			**Severe chronic GvHD**		
	**HR (95% CI)**	**P**		**HR (95% CI)**	**P**	
**Pre-conditioning Leptin (per log2)**	1.10 (0.90-1.33)	0.373		1.06 (0.76-1.48)	0.721	
**Age at transplant (per 10 years)**	1.01 (0.82-1.25)	0.919		0.67 (0.47-0.94)	0.023	*
**Disease stage[+]**						
Early	Ref	Ref		Ref	Ref	
Intermediate/Advanced	1.14 (0.66-1.97)	0.650		0.81 (0.31-2.15)	0.682	
**HLA match**						
match (10/10)	Ref	Ref		Ref	Ref	
mismatch	0.51 (0.23-1.15)	0.104		0.89(0.26-3.07)	0.857	
**Conditioning**						
MAC	Ref	Ref		Ref	Ref	
RIC	1.01(0.54-1.90)	0.966		2.16 (0.69-6.74)	0.186	
**ATG pre transplant**						
Not given	Ref	Ref	***	Ref	Ref	.
given	0.35 (0.20-0.60)	<0.001		0.43 (0.17-1.13)	0.087	
**Recipient sex**						
Female	Ref	Ref		Ref	Ref	
Male	1.18 (0.68-2.05)	0.553		0.92 (0.37-2.31)	0.863	
**Donor sex**						
Female	Ref	Ref		Ref	Ref	
Male	0.65 (0.37-1.16)	0.145		0.82 (0.31-2.15)	0.859	

*Significance code: '***' p< 0.001; '**' p<0.01; '*' p<0.05; '.' p<0.1; [+] According to Gratwohl et al., 2009 (34). Abbreviations: SIG-significance; HR-hazard ratio; HLA-human leukocyte antigen; RIC-reduced intensity conditioning; MAC-Myeloablative conditioning; ATG-anti-thymocyte globulin.*

Supplementary Table S10. Patient characteristics of the full Heidelberg cohort (discovery cohort).

**n**	524
**Year alloSCT performed**	2002-2013
**Median Age at alloSCT (range)**	54 (17 to 75)
**Recipient Sex**	
Female	205 (39%)
Male	319 (61%)
**Donor Sex**	
Female	168 (32%)
Male	356 (68%)
**Disease entity**	
AML	161 (31%)
ALL	45 (9%)
Other	318 (61%)
**HLA match**	
Match (10/10)	400 (76%)
Mismatch	124 (24%)
**Conditioning**	
MAC	90 (17%)
RIC	434 (83%)
**Pre-transplant ATG**	
Given	340 (65%)
Not given	184 (35%)
**Disease stage before alloSCT+**	
CR1	157 (30%)
CR2	133 (25%)
>CR2	215 (41%)
unknown	19 (4%)
**Serum Leptin Median in pg/ml (range)**	14,241 (529.56 to 97,994
**Serum Adiponectin Median in ng/ml (range)**	4,485.1 (747.9-20,000)
**Serum Vitamin D Median in ng/ml (range)**	11.9 (1 to 46.3)
**Body Mass Index Median in kg/m²**	24.7 (16.3 to 47.6
**% Change in Body weight 3 months prior to alloSCT compared to transplantation day Median (range)**	-1.2 (-19.3 to 26.1)

+ According to Gratwohl et al., 2009 (21). Abbreviations: HLA, human leukocyte antigen; RIC, reduced intensity conditioning; MAC, Myeloablative conditioning; ATG, anti-thymocyte globulin. CR- complete remission before alloSCT

Supplementary Table S11. Patient characteristics of the full Essen cohort.

**n**	367
**Year alloSCT performed**	2009-2013
**Median Age at alloSCT (range)**	55 (17 to 73)
**Recipient Sex**	
Female	189 (51%)
Male	178 (49%)
**Donor Sex**	
Female	122 (33%)
Male	245 (67%)
**Disease entity**	
AML	367 (100%)
**HLA match**	
match (10/10)	262 (71%)
mismatch	105 (29%)
**Conditioning**	
MAC	48 (13%)
RIC	319 (87%)
**Pre-transplant ATG**	
Given	213 (58%)
Not given	154 (42%)
**Disease stage before alloSCT$^+$**	
CR1	182 (50%)
CR2	97 (26%)
>CR2	88 (24%)
**Serum Leptin Median in pg/ml (range)**	9564 (91.62 to 92219)
**Serum Vitamin D Median in ng/ml (range)**	10.3 (0 to 39.2)
**Body mass index Median in kg/m^2 (range)**	25.7 (17.5 to 47.1)

$^+$ *According to Gratwohl et al., 2009 (21). Abbreviations: HLA-human leukocyte antigen; RIC-reduced intensity conditioning; MAC-Myeloablative conditioning; ATG-anti-thymocyte globulin. CR- complete remission before alloSCT*
